# On paper; in practice: measuring compliance with official pricing policies in a large field study of essential medicines in Indonesia

**DOI:** 10.1080/20523211.2025.2521434

**Published:** 2025-07-02

**Authors:** Ayu Rahmawati, H. U. Ramadaniati, Y. Anggriani, W. Nathanial, E. Pisani

**Affiliations:** Faculty of Pharmacy, Pancasila University, South Jakarta, Indonesia

**Keywords:** Medicine price, maximum retail price, price transparency, mark-ups

## Abstract

**Background:**

In 2015, Indonesia Ministry of Health (MoH) issued a decree to ensure the affordability of medicines by providing transparent prices which obliged all manufacturers to print a maximum retail price (MRP) on medicine primary packaging. We measure the compliance of manufacturers and retailers with the regulation stated in the decree and estimate the retailers’ profit.

**Methods:**

Five essential medicines (allopurinol, amlodipine, amoxicillin, cefixime, dexamethasone) were purchased from randomly selected health facilities, retail pharmacies and online outlets in Indonesia. We recorded retailer’s selling price and product’s MRP. We also sourced product’s list price and sales volume from pharmaceutical market data. We conducted an MRP policy implementation analysis by evaluating three indicators (retail price transparency, permitted MRP and permitted sales price). We also estimated the retailers’ profit by taking into account the profit margin and sales volume.

**Results:**

Of 1249 sampled medicines, nearly all samples (99.4%) had visible MRP on their packaging indicating high transparency compliance. For unbranded generics, none complied with permitted MRP with a median ratio of printed MRP to permitted MRP being 3.5 (IQR 2.6–7.9), whilst higher compliance (11.2%) was observed for branded generics (IQR 1.1–1.7, median 1.1). 33% of the samples were sold above the printed MRP with the lowest compliance to actual selling price being documented in hospitals. Branded generics accounted for 79% of the product value across the study medicines and generated more profits than the unbranded versions.

**Conclusion:**

The transparency implementation through printed MRP and the compliance with permitted sales price regulation contribute to retailer accountability. Rules restricting permitted MRPs for unbranded generics, irrational since their inception, while they never set any limits at all on the price of branded medicines. This showed not enough evidence that the policy contributed to its objective of ensuring affordability.

## Background

The World Health Assembly has repeatedly expressed concern about inequitable access to medicine, and financial hardship associated with high drug prices (World Health Assembly, 2019). In order to improve affordability, countries have implemented a variety of medicine price containment strategies, including increasing the use of unbranded generics; enforcing price transparency; setting maximum mark-ups or retail prices; and procurement using reference pricing and consolidated tenders (Vogler et al., 2017). In 2020, the World Health Organisation summarised the evidence for these in its guidelines for medicine pricing policies (World Health Organization, 2020). Only one of the 10 policies merited more than a conditional recommendation, illustrating the poor evidence available in this area. A more recent paper provides a framework for developing effective medicine pricing policies for low and middle-income countries (Babar, 2024). In this paper, we consider evidence from Indonesia, a middle-income country with the world’s fourth largest population, investigating how successfully policies regulating mark-ups and requiring price transparency restrict prices paid by patients across the archipelago.

### Medicine price transparency

Price transparency policies aim to increase accountability by promoting disclosure and dissemination of prices of pharmaceutical products to interested actors. WHO suggests that countries improve price transparency by disclosing prices along the supply chain and distribution chain and sharing the net transaction prices to relevant stakeholders (World Health Organization, 2020). The role that price transparency plays in promoting access to medicines is contested and inconclusive, precisely because such initiatives rarely encompass undisclosed rebates or discounts (Ahmad et al., 2020; Vogler & Paterson, 2017). In 2019, the World Health Assembly urged Member States to facilitate the sharing of health product prices with the public (World Health Assembly, 2019) and, in 2020, WHO conditionally recommended price transparency as a price control strategy in policy guidelines (World Health Organization, 2020). A 2019 report by the Office for Health Economics thinktank advocated for more transparency in procurement processes but argued that sharing prices of on-patent medicines would slow diffusion to lower-income settings, a view shared by other authors (Berdud et al., 2019; Danzon et al., 2005; Kyle & Ridley, 2007). For off-patent medicines with multiple suppliers, price transparency is considered to have greater potential as a tool to increase efficiency in public procurement, with the important proviso that measures must be in place to prevent supplier collusion (Berdud et al., 2019).

### Medicine price mark-up as a market incentive

The WHO pricing policy guidelines also consider regulation of mark-ups, the margins added to the price of medicines at every step of the supply chain (World Health Organization, 2020). A systematic review of pharmaceutical price mark-up policies identified several variants of mark-ups, as percentage of the price paid to the previous supplier, specific value, or a combination of both (Lee et al., 2021). The review also documented higher mark-ups in Asian countries (up to 50%) compared to Western countries (4% – 25%). Legislation restricting mark-ups to a fixed percentage usually aims to cap profit margins at different levels of the supply chain, and further to incentivise the provision of specific products or supply to remoter areas by allowing higher margins for products and destinations that would otherwise not be considered commercially viable. The WHO pricing guidelines conditionally recommend regulating mark-ups on medicines principally as a way of securing access, while guarding against excessive out of pocket spending. Notably, WHO warned against fixed percentage mark-up restrictions, which disincentivise the provision of cheaper medicines and brands (World Health Organization, 2020).

### Medicine price regulation in Indonesia

In 2014, Indonesia initiated a mandatory national health insurance scheme (Jaminan Kesehatan Nasional/JKN). By 2024, it had 273 million members, 97% of the population (Social Security Agency on Health, 2024). JKN is intended to cover the cost of both care and medicines. However, supply side constraints, bureaucratic hurdles and brand preference have led many Indonesians to continue buying their own medicines from health facilities, pharmacies or over the counter. The percentage of out of pocket (OOP) health expenditure was still relatively high at 27.5% in 2021 (when 86% of the population was registered with JKN) (Social Security Agency on Health, 2022; World Health Organization, 2024).

#### Ceiling prices for public procurement

At the time of this study, the Indonesian government held provincially consolidated tenders for essential medicines. Any company with a valid market authorisation could bid. The lowest bidder under a government-set ceiling price won the right to supply for one or more provinces. The winning companies and tender prices were published in a publicly accessible electronic catalogue (e-catalogue), through which healthcare facilities ordered medicines. However, the ceiling price, and clear information about how it was set, was not disclosed publicly.

#### Maximum retail price policy

In 2015, Ministry of Health (MoH) Decree No 98 obliged all medicine manufactures to print a maximum retail price (MRP) on the primary packaging of every medicine. The stated aim of this rule was to protect the affordability of medicines by providing transparent price information to the public about the acceptable price of medicines they bought out of pocket (Minister of Health Regulation Number 98 of, 2015 Concerning Provision of Information on the Highest Retail Price of Drugs, 2015). Permissible MRPs were regulated as follows:

- Any unbranded generic version of a medicine (active ingredient, dose and formulation) that had a winning tender price listed in e-catalogue could retail for 1.28 times the published provincial public procurement price.
- All unbranded medicines that had no version listed in e-catalogue, as well as all medicines sold under a brand name (both generic and originator), could retail for up to 1.28 times the list price (also known as the net pharmacy price), which can be set by the market-authorisation holder at any level.

The 28% margin was designed to include a 10% value-added tax (VAT) and an 18% profit margin (pharmacy service fee). The regulation states that no pharmacy or over-the-counter medicine seller may charge more than the MRP printed on the packaging, except if the price is no longer in accordance with applicable provisions.

Our study investigates the level of compliance by manufacturers and retailers with these regulations. We further estimate retailers’ profits on unbranded and branded medicines.

## Methods

### Study design

This study forms part of a larger study of medicine quality in Indonesia known as STARmeds. STARMeds methods are described in detail elsewhere (STARmeds Study Team, 2023). Briefly, we purposively chose seven sampling areas to reflect Indonesia’s geographic, economic and demographic diversity. These were the Greater Jakarta region, and a large city and a more remote rural district in each of Western, Central and Eastern Indonesia (respectively Medan/Labuhan Batu; Surabaya/Malang; Kupang/Timor Tengah Selatan). Within each area, we listed, verified and randomly selected outlets (pharmacies; over-the-counter medicine shops; hospitals; health centres; private doctors, and midwives). We also listed online platforms that sell medicine. Professional data collectors trained as mystery shoppers bought 1003 samples of five common medicines (amlodipine, amoxicillin, allopurinol, cefixime, and dexamethasone) at retail outlets and online, and recorded the purchase price. Because we used the mystery shopping approach, we did not provide informed consent to the staff of the study retail outlets. Ethics committees approve of this approach. Retail outlets were visited by two shoppers on different days; each signalled their desire for a specific price point (‘better quality’ or ‘more affordable’ versions of the target medicine). We bought overtly and/or got free another 246 samples from health facilities and health providers.

### Data analysis

#### 1) Price along the medicine value chain

**Figure 1.**
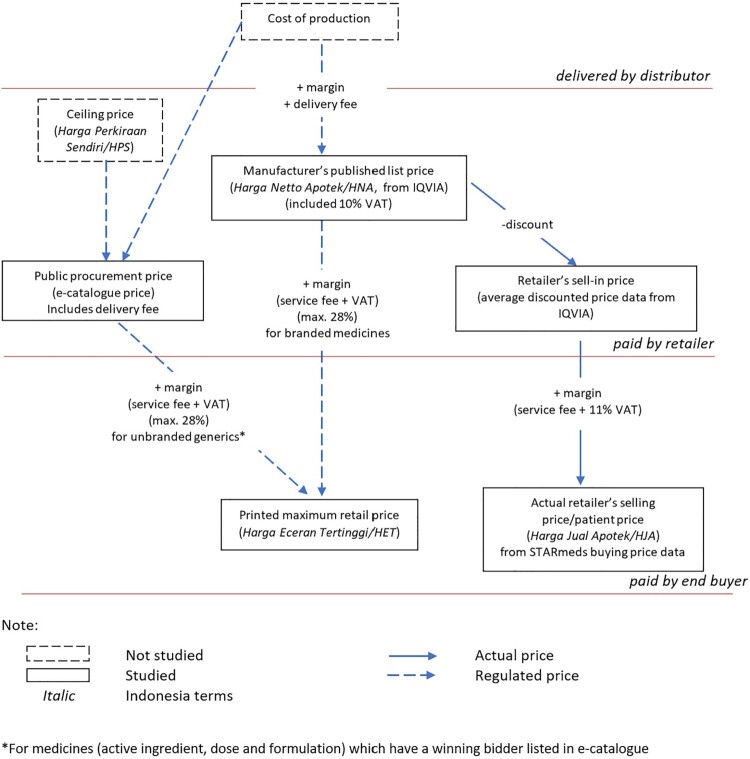
Schematic illustration of prices in Indonesia’s pharmaceutical market.

This paper investigated actual prices at different points in the supply chain in Indonesia, comparing them with prices permitted by regulation. Figure 1 provides a schematic illustration of actual and permitted or notional prices; more detailed definitions and data sources are provided in Supplemental Table S1.

In this study, we analysed the price along the value chain for 5 medicines: allopurinol (100 and 300 mg oral tablet), amlodipine (5 mg oral tablet), amoxicillin (500 mg oral tablet or capsule and 125 mg/5 mg dry syrup), cefixime (100 mg oral tablet or capsule), and dexamethasone (0.5 mg oral tablet). From STARmeds’ study conducted in Feb–May 2022, we gathered the price actually paid for each sample, and their printed MRP. We dropped 87 samples provided free by health facilities and health providers for any analysis related to the actual selling price.

A total of 7 samples did not have legible maximum retail price information. We imputed them using maximum retail price information for the same product with the nearest expiry date. The price of public procurement was based on the maximum provincial public procurement price applicable in 2022. We bought the 2022 manufacturer’s published list price, retailer’s sell-in price (usually discounted price), and sales volume quarterly by type of outlets for each product (medicine with unique dosage, formulation, and brand) from IQVIA, a pharmaceutical market company. A total of 20 products (covering 35 samples) that we collected in our field survey did not appear in IQVIA’s product listings. For these products, we imputed missing data as follows:

- List price = MRP × the mean ratio of the list price to the MRP, by active ingredient
- Retailer’s sell in price = List price × the mean ratio of retailer’s sell in price to list price, by active ingredient
- Sales volume: Described in detail in the STARmeds archive (Pisani & Rahmawati, 2023)

#### 2) Policy implementation analysis

In the period when the study was conducted, Ministry of Health Decree No. 98 of 2015 was in force. Table 1 describes elements of the regulation and defines the indicators we use to investigate the extent to which samples and products in our study complied with the decree. Logistic regression analysis was conducted to identify interactions between factors correlated with retailers’ non-compliance with permitted sales price regulation (overcharging practices).

**Table 1.** Elements of 2015 decree regulating pricing and transparency and non-compliance indicators.

Policy Regulation	Indicator	Non-compliance indicator
Maximum retail price must be clearly and legibly printed in indelible ink on the primary packaging of all medicines.	Retail price transparency	$=\frac{\mathrm{Number}\;\mathrm{of}\;\mathrm{samples}\;\mathrm{with}\;\mathrm{no}\;\mathrm{clearly}\;\mathrm{printed}\;\mathrm{MRP}\;}{\mathrm{Total}\;\mathrm{number}\;\mathrm{of}\;\mathrm{samples}}\times 100\text{\%}\;$
The permitted MRP for unbranded medicines* is the provincial e-catalogue price plus 28%. The permitted MRP for all other medicines is the list price plus 28%.	Permitted MRP	$=\frac{\mathrm{Number}\;\mathrm{of}\;\mathrm{individual}\;\mathrm{products}\;\mathrm{with}\;\mathrm{printed}\;\mathrm{MRP}\;>\mathrm{permitted}\;\mathrm{MRP}\;}{\mathrm{Total}\;\mathrm{number}\;\mathrm{of}\;\mathrm{individual}\;\mathrm{products}\;(\mathrm{by}\;\mathrm{API},\;\mathrm{dose}\;\mathrm{formulation})}\;\times 100\text{\%}$
Outlets are not allowed to sell medicine above the printed MRP.	Permitted sales price	$=\frac{\mathrm{Number}\;\mathrm{of}\;\mathrm{samples}\;\mathrm{with}\;\mathrm{sales}\;\mathrm{price}\;>\;\mathrm{to}\;\mathrm{printed}\;\mathrm{MRP}}{\mathrm{Total}\;\mathrm{number}\;\mathrm{of}\;\mathrm{samples}}\times 100\text{\%}$

MRP: Maximum retail price

*For medicine (active ingredient, dose and formulation) which have a winning bidder listed in e-catalogue

#### 3) Profit estimation

We estimated the profit on each product in our study by subtracting VAT from the median of actual selling price, then subtracting the retailer’s sell-in price for the product obtained from distributor that usually has been discounted. This weighted average discounted price is usually lower than the list price. We estimated the total profit retailers made in the Indonesian market by multiplying the profit for each product by its 2022 sales volume and summing the total profits of those products. We compared profits across medicines and between unbranded and branded medicines.

## Results

### Policy implementation analysis

#### 1. Implementation of maximum retail price transparency

**Figure 2.**
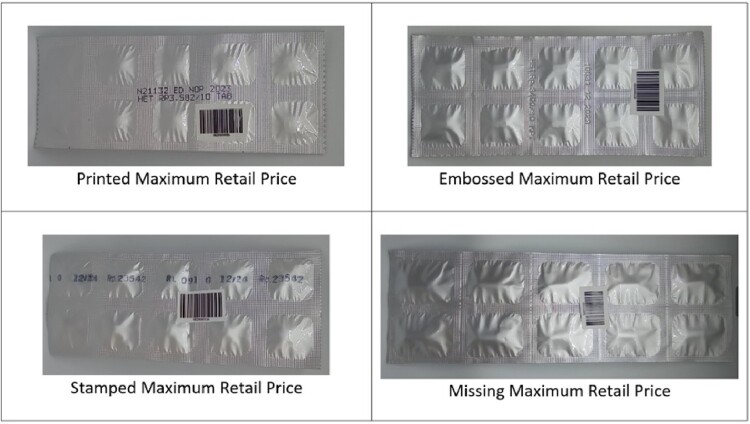
Examples of maximum retail price visibility.

Compliance with the retail price transparency regulation was 99.4%. Just 7/1249 samples collected in the field had no visible MRP data on their primary packaging. All were branded medicines; 2 were illegally imported versions of products registered in Indonesia, and 1 consisted of incomplete strips of medicine. Manufacturers printed the MRP on the primary packaging using various formats including inkjet printing (70.7% of unique products in our study), stamping (24.4%), and embossing (4.9%). The printed form is usually readable and hard to erase, stamped is readable and erasable, and embossed is hard to read but non-erasable. Some examples of the printed MRP visibility can be seen in Figure 2. Products with embossed MRPs generally had lower MRP than those where data were stamped or printed (the median ratio of embossed MRP to the lowest MRP by medicine is 3.8 vs 4.4 for printed MRP vs 5.7 for stamped MRP).

#### 2. Non-compliance with permissible MRP regulation

Non-compliance with permissible MRP regulation differed between unbranded generics benchmarked to the e-catalogue price and other medicines. We collected 80 unique unbranded generic medicines with 98 different printed MRPs. All of them had printed MRP above the maximum permissible retail price, for a non-compliance rate of 100%. This was true even for the products that were winners of the public procurement tenders (and thus achieved significant economies of scale). Table 2 lists the public procurement price of the winning products, the maximum permissible retail price according to the regulations, and the actual MRP printed on the packaging of public procurement winner products. The printed MRP for public procurement medicines was from 1.3–16.6 times the permissible MRP. In the latter case, samples with a later expiry date had a lower MRP, suggesting that the manufacturer lowered the printed price over time, though the lower price was still 5.9 the permitted maximum.

**Table 2.** E-catalogue price and printed MRP for public procurement winner products.

Medicine	Dosage and formulation	Authorisation holder of the e-catalogue products (masking company names)	E-catalogue price	Permitted MRP for based on regulation	Printed MRP based on STARmeds’ samples	Ratio between actual and permitted MRP
Allopurinol	100 mg tablet	Company A	99	127	333	2.6
Allopurinol	300 mg tablet	Company A	215	275	700	2.5
Amlodipine	5 mg tablet	Company B	61	78	462; 1296	5.9; 16.6
	5 mg tablet	Company C	59	76	No sample	.
	5 mg tablet	Company D	53	68	288	4.2
Amoxicillin	125 mg dry syrup	Company C	204	261	340; 416	1.3;1.6
Amoxicillin	500 mg tablet	Company A	251	321	463	1.4
Cefixime	100 mg tablet	Company E	409	524	3233	6.2
	100 mg tablet	Company A	398	509	1788	3.5
Dexamethasone	0.5 mg tablet	Company F	38	49	154	3.2
	0.5 mg tablet	Company G	35	45	99	2.2

**Figure 3.**
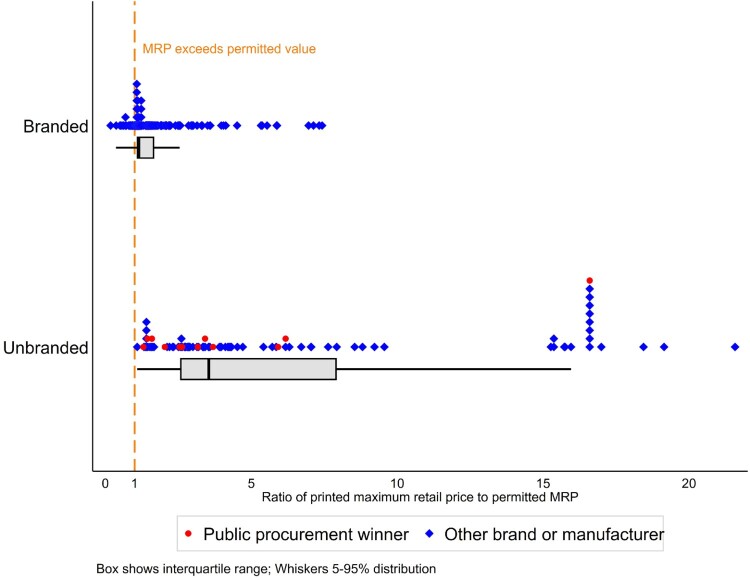
Ratio of printed maximum retail price (MRP) to maximum permissible retail price, by branded status.

For branded medicines, market authorisation holders are free to set the list price at whatever they feel the market will bear. The 2015 pricing decree sets the maximum permissible retail price for branded medicines at the market-authorisation holder’s chosen list price, plus a margin of 28% (which includes VAT). We collected 154 unique branded generic medicines with 205 different printed MRPs. While just 11.2% of branded products technically complied with the maximum permissible retail price legislation, most deviated by only a small difference, compared with unbranded medicines. The median ratio of printed to permitted MRP’s for branded medicines was 1.1 (IQR 1.1–1.7.), compared with 3.5 for unbranded generics (IQR 2.6–7.9, *p* < 0.00). The significantly different distribution is shown in Figure 3.

All but one of the 20 products that had printed MRPs over 10 times the permitted amount was unbranded amlodipine 5 mg tablets. This cluster accounted for 18/27 unbranded amlodipine samples studied. Though compliance with MRP regulations among branded products is relatively good, with over 50% complying or exceeding permitted MRPs by less than 10%, the real variation in list prices is vast. If we compared the MRP for both unbranded and branded products with the lowest MRP of each medicine with the same dose and formulation, the ratio ranged up to 67.5 times (IQR 2.7–9.1, median 4.5).

#### 3. Retailer compliance with maximum retail price

The pricing and transparency decree of 2015 states that pharmacies may not charge patients more than the MRP printed on the packaging. Flexibility is provided in the decree – it does not apply if the printed price ‘is no longer in accordance with applicable provisions’. However, health facilities selling at prices above the MRP must provide reasons to patients, as well as alternative unbranded generics if available.

Not only the MRP but also the actual retailer’s selling price of the product for the same medicine demonstrated wide variation. The median selling price of branded medicine was consistently higher than the unbranded one. It can be seen from Supplemental Table S2 that the highest ratio of the most expensive branded generic to the cheapest unbranded counterpart is observed for Amlodipine 5 mg tablet, with ratio of 173 times. Meanwhile, the other study medicines had varied ratios within the range of 13.0-86.8. However, it is important to note that for Dexamethasone 0.5 mg tablet, the lowest price was found in one of the branded generics.

**Figure 4.**
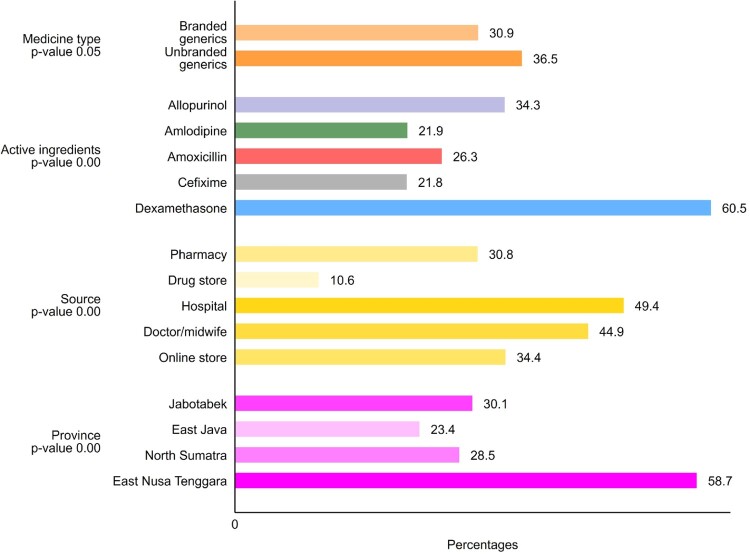
Percent of samples with patient price more than the maximum retail price.

Most retailers charged below the MRP; the median price paid for a medicine in the study was 88% of the maximum permitted price. Our mystery shoppers or (for hospitals and health care providers) overt sample collectors were charged more than the printed MRP for 33.1% of the 1162 samples that they bought. However, overcharging was rarely extreme; the median excess among overcharged samples was 26% of the permitted price (IQR 7.5–72%), which amounts in real terms to a median of 141 rupiahs per tablet/unit; less than one-tenth of a US cent. As Figure 4 indicates, overcharging appeared more common for unbranded than branded generics (36.5 vs 30.9%, *p* = 0.05). Hospitals were most likely to overcharge for medicines, with private doctor/midwife ranking second and online store ranking third. Buyers in East Nusa Tenggara, in the remoter eastern region of Indonesia, were also more likely to be charged more than regulations allowed.

We included all those factors in a logistic regression model to identify interactions between factors correlated with overcharging. As detailed in Table 3, after controlling for differences in medicines and sales location, patients were 66% more likely to be overcharged for unbranded than for branded medicines. Overcharging was most common for dexamethasone; 4.0 times higher than for the reference medicines allopurinol. However, because this was the cheapest unit-cost medicine in the study, the mean amount of the overcharge, at 162.5 rupiah, was significantly lower than the mean for other medicines (485.5, *p* < 0.000). Outlet type had little effect, except for hospital where overcharging was likely found, and the model confirmed the high relative prices paid in Eastern Indonesia; patients were 2.7 times more likely to be overcharged there, compared with Jakarta (the country’s capital), after controlling for other factors.

**Table 3.** Logistic regression results of factors that correlated with overcharging.

Variables	Odds Ratio (*p*-value)
**Brand status**	
Unbranded generics	1.663 (0.001)
Branded generics (ref)	
**Active ingredients**	
Amlodipine	0.495 (0.001)
Amoxicillin	0.722 (0.098)
Dexamethasone	3.997 (0.000)
Cefixime	0.505 (0.003)
Allopurinol (ref)	
**Source**	
Drug store	0.288 (0.014)
Hospital	2.127 (0.004)
Doctor/midwife	1.786 (0.032)
Online store	1.290 (0.149)
Pharmacy (ref)	
**Province**	
East Java	0.478 (0.000)
North Sumatra	0.764 (0.162)
East Nusa Tenggara	2.687 (0.000)
Jakarta (ref)	
**Constant**	0.379 (0.000)
**Number of observations**	1162
**Pseudo R^2^**	0.138 (0.000)
The category (ref) was set as reference group	

### Profit estimation

**Figure 5.**
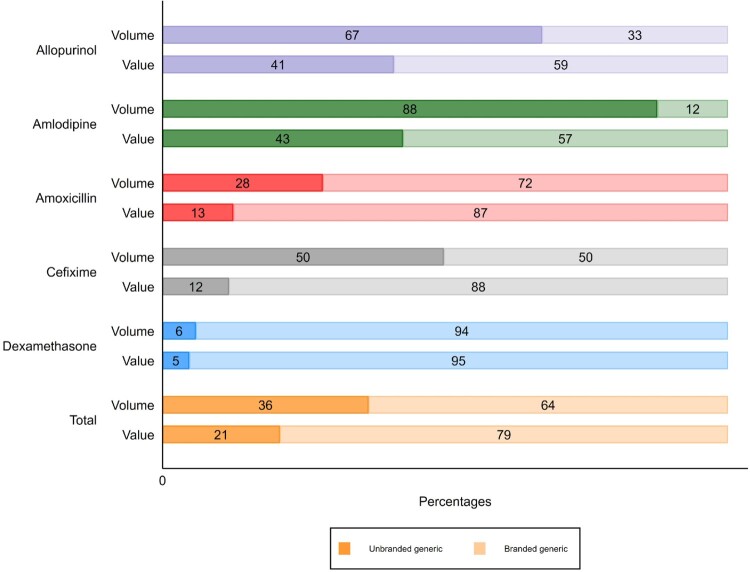
Estimated sales volume and value in percentages, by medicine.

To estimate the absolute retail value of each unique product (by active ingredient, dose and formulation) in the Indonesian market, we multiplied the median actual selling price for the product by its sales volume. Products that were collected and bought during STARmeds sampling represent 94.6% of IQVIA sales volume data. When aggregated at the level of the medicine, two of the five study medicines sold more units of unbranded than branded products (Allopurinol and Amlodipine). However, when calculated by value, branded sales outstripped unbranded sales in every case, as shown in Figure 5. Across all study medicines, branded products accounted for 64% of volume, but 79% of value.

Many pharmaceutical companies sell to pharmacies, hospital, and, especially, drug store at prices lower than the list price. When we compared the price that we paid for each sample with the retailer’s sell-in price charged by distributors for the same brand and dose, we found a median margin of 96.2%. For unbranded generics, the median was 88.8% (IQR 29.9–192%), while for branded medicines it was higher, at 103.8% (IQR 46.3–207.44%). We further estimated profits on each unique product in the Indonesian market as described in the methods. Overall estimated profits, at $USD 4,474 thousand, were 3.1 times higher on branded than on unbranded products. Only for hypertensive medicine amlodipine did estimated profits on unbranded products outstrip profits on brands (Table 4).

**Table 4.** Estimated retailer’s profit of study medicine.

Medicine	Medicine Type	Median unit price in IDR	Median profit margin in %	Total sales volume (smallest units)	Estimated total profits in IDR	Estimated total profits in USD
Allopurinol	Branded	1,411	42.2	9,274,437	5,343,954,295	359,790
	Unbranded	400	46.1	18,933,688	4,170,092,088	280,758
Amlodipine	Branded	3,358	22.7	3,843,826	2,886,251,657	194,321
	Unbranded	500	55.4	26,919,356	7,300,350,418	491,507
Amoxicillin	Branded	750	45.9	28,244,160	16,933,351,233	1,140,063
	Unbranded	500	35.1	11,166,353	2,492,931,785	167,840
Cefixime	Branded	17500	37.4	2,647,191	14,118,966,145	950,580
	Unbranded	1892	39.3	2,619,275	1,853,682,865	124,802
Dexamethasone	Branded	250	55.1	67,400,432	10,794,327,827	726,744
	Unbranded	225	68.8	4,222,152	555,691,380	37,413

*Average exchange rate for 2022: US$1 = 14,853 IDR

## Discussion

### Policy implementation analysis

Although Indonesia set out regulations to enforce price transparency with the goal of increasing the affordability of medicines paid for out of pocket nearly a decade ago, compliance with these policies has not, to our knowledge, been assessed. We used mystery shoppers to buy samples of a wide variety of brands of 5 common essential medicines to measure the extent to which market authorisation holders and medicine retailers follow in practice the provisions in force on paper.

We found that a requirement to print a maximum retail price on primary packaging was almost universally observed, although the embossed data common on cheaper products did not always meet the ‘easily readable’ requirement.

Market authorisation holders did not, however, comply with restrictions on the level of that maximum retail price, with printed MRPs ranging up to 16.5 times higher than the permitted MRP, and actual MRPs ranging up to 67.5 times as high compared to the lowest MRP for the same medicine, dose and formulation. For unbranded generics, non-compliance was 100%. This is most likely an indication of poorly thought-through policy making, rather than venal behaviour on the part of pharmaceutical companies. The regulations set as a benchmark price the lowest tender price bid by manufacturers which benefit from massive economies of scale, and near-zero marketing and transaction costs. Studies have questioned the level of winning tender prices, (Anggriani et al., 2020; Kristina et al., 2020), arguing that they may be too low to remain sustainable, and may thus threaten supply. Certainly, it is unreasonable to expect all other manufacturers of unbranded products to effectively match nationally consolidated procurement prices in their wholesale supplies to much smaller retail markets.

Other aspects of the policy are also irrational: for example, it allows for different MRPs by province, while pharmaceutical firms with central factories cannot feasibly print different MRPs and selectively distribute them to different provinces. Medicine expiry dates were not aligned with changes in e-catalogue prices, so MRPs that had previously been compliant may become non-compliant during the product’s shelf-life. Finally, the provision has remained in force even after the public procurement system moved away from a single-price system, removing the possibility of benchmarking.

Compliance or near-compliance with permitted MRPs was much more common for branded products: 50% of samples were below or within 10% of the permitted MRP. This is also to be expected, since the benchmark price, in this case, is set by pharmaceutical companies themselves, at any level they believe the market will bear (Wasir et al., 2019). For these products, the MRP cannot claim to act as a price constraint for pharmaceutical companies. It does, however, allow the discerning customer to compare brands.

The visible MRP was also intended to ‘increase accountability’ and thus guard against overcharging. In practice, 33% of the retailers selling medicines to patients buying medicines out of pocket overcharged for them. This mirrors another Indonesian study where 21% of five cardiovascular and diabetes medicines were sold above the MRP (Pisani et al., 2023). We found overcharging in most of all types of outlets (except public primary health where patient got medicine for free that we dropped for the analysis) and all study regions, noting, however, that it was most common for the cheapest medicines. Overcharging was more common in Eastern Indonesia, probably less a sign of pharmacist greed than of a policy that does not feasibly account for market realities. In practice, products all bearing the same MRP are distributed nationally. However, Indonesia’s geography – over 13,000 islands – means that distribution costs to the sparsely-populated East are high. Retailers in islands remote from the centres of production pass these costs on to consumers.

Evidence on maximum retail price enforcement in other middle-income settings is limited. Efforts in the Philippines to restrict prices of specific medicines through regulation of wholesale and retail prices in 2020 (President of the Philippines Executive Order No 104 of, 2020 on Improving Access to Healthcare through the Regulation of Prices in the Retail of Drugs and Medicine, 2020) have not yet been fully evaluated, although a Ministry of Health spokesperson noted that effective implementation of the regulation would require flexibility and regular updating of price caps, something which has proven difficult to achieve in practice in the context of MRPs linked to public procurement prices as regulated in Indonesia. In 2004, South Africa moved to cap out-of-pocket prices in the private sector by requiring pharmaceutical companies to agree on a single, published price for listed prescription medicines (Department of Health of South Africa, 2004). This approach, which stands in contrast to Indonesia’s policy allowing every company to set prices for branded medicines, pushed prices for patients down, but the tenacity of the policy suggests that it did not substantially damage service provider sustainability (Moodley & Suleman, 2019, p. 2019).

How the benchmark price was set in the regulation was irrational for the unbranded products and not transparent enough for branded products, resulting unpredictable price charged to the Indonesia patient as indicated with widely varying range prices for the same medicine. The government should be clear about the purpose of the MRP regulations. If they are designed primarily to facilitate patient choice and to protect against excessive mark-ups by hospitals and pharmacies, then the government simply needs to dedicate more resources to ensuring compliance by retailers with current rules. In addition, if however, the intention is to ensure affordability by limiting the absolute price of medicines, then additional measures, such as the imposition of national ceiling prices, may be needed. As Babar (2024) suggests the development of pharmaceutical policies needs to be locally tailored, transparent, stable, and predictable.

Our work, as well as a more detailed analysis of affordability carried out by our research group, Maria et al. (2024), suggests that at present, affordable versions of many essential medicines are universally available in the Indonesian market. This suggests that competitive pressures in the market currently ensure that Indonesian patients at all income levels can already access these common medicines. In these cases, further price setting is not needed. However, there may be other countries with fewer manufacturers, for which government intervention in prices set by manufacturers may be warranted.

### Profit margin estimation

While the MRP regulation caps retailers’ mark-ups at 18% above the list price, the margins they earn in practice are based on the (often much lower) discounted prices at which they buy medicines from distributors. By this measure, we estimated median mark-ups at 96.2%, excluding VAT. Since the mark-ups measurement may be different in previous research in this area, it is difficult to make a comparison with other studies. Health Action International’s study in Kenya documented mark-up as the percentage between patient price and procurement price. In private health facilities, the mark-up in the range of 228% for imported medicines and 376% for local products compared to procurement price, far exceeding the regulated rate of 18%, meanwhile in public health facilities, the mark-ups is 35% for imported medicines and 177% for local product (Ewen & Okemo, 2018).

Meanwhile, in India, researchers recorded retailers’ mark-up as the percentage between MRP and price to retailer (PRT) (Singal et al., 2011). Mark-ups for unbranded medicines in the range of 201–1016% compared to their maximum retail price, much higher than the 16–20% permitted. Overcharging was lower for branded medicines, for which observed mark-ups were in the range of 25–30%.

Though not in itself the object of one of the investigated policies, we include an analysis of absolute profits because they define the success of a retail pharmacy, and influence the way pharmacists respond to existing regulations. Absolute profits are determined by profit margins in combination with base price and sales volume. Indeed, WHO recommends against fixed-percentage-based mark-up policies such as those implicit in Indonesia’s MRP policies and espoused in the examples above, because they disincentivise the sale of products with a relatively low base price (World Health Organization, 2020)

Amlodipine, the only cardiovascular medicine in the study, was the only one for which profits were greater on unbranded products. This is consistent with findings reported by Pisani et al. (2023) in a study of cardiovascular and diabetic medicine in East Java. In that study, unbranded generics sold in much higher volumes and with higher profit margins than branded versions, generating more absolute profits.

We should note that our estimates of mark-ups and profits suffer because they are not based on the actual sell-in price to the specific pharmacy where we were buying samples, but on the weighted average discount for that brand nationwide during the period of the study. In practice, discounts can vary substantially between outlets and over time. However, we have no reason to believe that these temporal variations would differ between study medicines or by branded status, meaning that our comparative conclusions are likely robust. We were also obliged to impute values for a small proportion of samples, as described in the methods.

## Conclusion

Virtually all medicines in Indonesia display a maximum retail price on their packaging. This probably limits extreme price gouging at pharmacies. While shoppers were charged over the MRP in more than 30% of our transactions, fewer than 5% paid over twice the permitted price for a medicine and those that did were mostly buying low-priced unbranded medicines. Higher ‘overcharging’ in more remote areas suggests that some of the higher prices may have been a fair response by retailers to higher costs, in areas where the population is well accustomed to paying higher prices for commodities.

Transparency may, thus, contribute to retailer accountability. Although ensuring affordability is given as one goal in the preamble to the regulation on maximum retail pricing, affordability is not defined. Further, the policy itself is not well suited to achieving this aim, because it allows producers to set the price for branded products (including branded generics) at any level of their choosing. Rules restricting MRPs for unbranded generics, irrational since their inception, can no longer be implemented at all following changes to the public procurement rules (National Procurement Agency Regulation No 9 of, 2021 on online merchants and electronic catalogue in procurement of good/service in public sector, 2021). Whether more effective regulation of the absolute level of MRP should be introduced or not is debateable in the context.

## Supplementary Material

Supplemental Material Table S1

Supplemental Material Table S2

## Data Availability

Masked version of STARmeds data is available in the STARmeds repository: https://dataverse.harvard.edu/dataverse/STARmeds (doi: 10.7910/DVN/RKYICP). Other data has confidential agreement. Further inquiries can be directed to the corresponding authors.
