# Supplementary material for: On paper; in practice: measuring compliance with official pricing policies in a large field study of essential medicines in Indonesia
Source: J Pharm Policy Pract. 2025 Jul 2;18(1):2521434. doi: 10.1080/20523211.2025.2521434 (PMC12224725; doi:10.1080/20523211.2025.2521434)
Supplement: Supplemental Material Table S1 [file JPPP_A_2521434_SM2356.docx]

Supplementary Table 1. Definition and data source of various type price

| **English name** | **Indonesian name** | **Definition** | **Source of data** | **Regulations** | **Notes** |
| --- | --- | --- | --- | --- | --- |
| Actual retailer’s selling price | Harga Jual Apotek (HJA) | Selling price from pharmacy to end users/patient | STARmeds’ field data (collected in Feb-May 2022) | - Actual retailer’s selling price should be below the printed maximum retail price | - 1126 records, one record per sample. - Different outlets or locations may sell the same product at different prices. - Includes 10% VAT charged from outlet to buyer |
| Printed maximum retail price (MRP) | Harga Eceran Tertinggi (HET) | Maximum retail price printed by industry on the primary packaging | STARmeds’ field data (collected in Feb-May 2022) | - Maximum retail price should not exceed the provincial e-catalogue price plus 28% margin for INN generic medicine; or the nett pharmacy price plus 28% margin for branded medicine | - 303 records, one record per product, per unique maximum retail price - Same products with different expiry dates may have different printed MRP - Includes 10% VAT charged from outlet to buyer |
| Market authorisation holders published  list price | Harga Netto Apotek (HNA) | Price set by market authorisation holder | IQVIA-local audit (2022) | Unregulated | - 5,196 records of study medicine, one record per product, per pack, per outlet type, per quarter - Includes 10% VAT charged from manufacturer to outlet |
| Average discounted price | N/A | Average price at which distributor sells to outlets after discount. | IQVIA-local audit (2022) | Unregulated | - 5,196 records of study medicines, one record per product, per pack, per outlet type, per quarter - Excludes any discounts - Different outlet types may have different discount prices for the same product - The weighted average discount price by volume is imputed if outlet type has missing price data |
| Public procure-ment price | Harga e-catalogue | Published procurement price for each province | Indonesian national procurement agency LKPP (procured in 2021 and applicable for 2021-2022) | - Prices for the same product are permitted to vary up to 20% between provinces to account for different distribution costs. | - 170 records of study medicines, one record per province - At the time of research, e-catalogue tenders were awarded to the single lowest bidder in each province - Includes distribution fee |
