# Supplementary material for: On paper; in practice: measuring compliance with official pricing policies in a large field study of essential medicines in Indonesia
Source: J Pharm Policy Pract. 2025 Jul 2;18(1):2521434. doi: 10.1080/20523211.2025.2521434 (PMC12224725; doi:10.1080/20523211.2025.2521434)
Supplement: Supplemental Material Table S2 [file JPPP_A_2521434_SM2355.docx]

Supplementary Table 2. Descriptive statistics of retailer’s selling price, by medicine

|  | | **Number of samples** | **Number of products** | **Retailer’s Selling Price** | | | | **Ratio of highest price of branded to lowest price of unbranded** |
| --- | --- | --- | --- | --- | --- | --- | --- | --- |
|  |  |  |  | **Mean** | **Median** | **IQR** | **Min-Max** |  |
| Allopurinol 100 mg tablet | Brand | 106 | 19 | 1394 | 995 | 400-2600 | 170-4250 | 40.9 |
|  | Unbranded | 107 | 16 | 405 | 307 | 267-500 | 104-1751 |  |
| Allopurinol 300 mg tablet | Brand | 42 | 17 | 2820 | 2800 | 807-3971 | 500-8950 | 37.3 |
|  | Unbranded | 34 | 5 | 623 | 598 | 500-700 | 240-1751 |  |
| Amlodipine 5 mg tablet | Brand | 90 | 28 | 4525 | 3630 | 1000-7850 | 90-14550 | 173.2 |
|  | Unbranded | 120 | 25 | 631 | 500 | 370-800 | 84-2481 |  |
| Amoxicillin 125 mg/5 ml dry syrup | Brand | 45 | 12 | 1037 | 667 | 442-1194 | 375-2833 | 13.0 |
|  | Unbranded | 16 | 6 | 521 | 500 | 417-646 | 217-833 |  |
| Amoxicillin 500 mg tablet | Brand | 141 | 32 | 2237 | 1300 | 700-4000 | 400-6100 | 20.9 |
|  | Unbranded | 72 | 7 | 642 | 515 | 495-700 | 292-1600 |  |
| Cefixime 100 mg capsule | Brand | 83 | 21 | 16522 | 21800 | 3833-27450 | 1000-40267 | 86.8 |
|  | Unbranded | 91 | 6 | 1947 | 1870 | 1200-2500 | 464-5000 |  |
| Dexamethasone 0.5 mg tablet | Brand | 186 | 24 | 413 | 300 | 200-450 | 62-1650 | 13.8 |
|  | Unbranded | 29 | 9 | 272 | 225 | 195-300 | 120-526 |  |

^*^Capsule was recoded as tablet
